# Retrosplenial and subicular inputs converge on superficially projecting layer V neurons of medial entorhinal cortex

**DOI:** 10.1007/s00429-022-02578-8

**Published:** 2022-10-14

**Authors:** Øyvind Wilsgård Simonsen, Rafał Czajkowski, Menno P. Witter

**Affiliations:** 1 Centre for Neural Computation, Egil and Pauline Braathen and Fred Kavli Centre for Cortical Microcircuits, Faculty of Medicine and Health Sciences, Kavli Institute for Systems Neuroscience NTNU Norwegian University of Science and Technology, Trondheim, Norway; 2 Nencki Institute of Experimental Biology, Warsaw, Poland

**Keywords:** Input convergence, Intrinsic circuitry, Entorhinal cortex, Hippocampal-entorhinal projections, Neuroanatomical tracing, Confocal microscopy

## Abstract

The medial entorhinal cortex (MEC) plays a pivotal role in spatial processing together with hippocampal formation. The retrosplenial cortex (RSC) is also implicated in this process, and it is thus relevant to understand how these structures interact. This requires precise knowledge of their connectivity. Projections from neurons in RSC synapse onto principal neurons in layer V of MEC and some of these neurons send axons into superficial layers of MEC. Layer V of MEC is also the main target for hippocampal efferents from the subiculum and CA1 field. The aim of this study was to assess whether the population of cells targeted by RSC projections also receives input from the hippocampal formation and to compare the distribution of synaptic contacts on target dendrites. We labeled the cells in layer V of MEC by injecting a retrograde tracer into superficial layers. At the same time, we labeled RSC and subicular projections with different anterograde tracers. 3D-reconstruction of the labeled cells and axons revealed likely synaptic contacts between presynaptic boutons of both origins and postsynaptic MEC layer V basal dendrites. Moreover, these contacts overlapped on the same dendritic segments without targeting specific domains. Our results support the notion that MEC layer V neurons that project to the superficial layers receive convergent input from both RSC and subiculum. These data thus suggest that convergent subicular and RSC information contributes to the signal that neurons in superficial layers of EC send to the hippocampal formation.

**Supplementary Information:**

The online version contains supplementary material available at 10.1007/s00429-022-02578-8.

## Introduction

The hippocampal formation and the parahippocampal region (HF and PHR, respectively) form a brain network essential for spatial memory and navigation. Place cells in HF were shown to represent specific locations within an environment (O'Keefe and Dostrovsky 1971; Wilson and McNaughton 1993). Subsequently, neurons that track head direction were reported as a prominent functional cell type in the dorsal presubiculum (Ranck 1985; Taube et al. 1990a, b; Taube et al. 1990a, b) and cells that encode position, head direction, borders, and speed were found in the medial entorhinal cortex (MEC; Fyhn et al. 2004; Hafting et al. 2005; Sargolini et al. 2006; Solstad et al. 2008; Kropff et al. 2015). Inactivation or lesioning of the MEC leads to remapping, unstable place fields and, in certain cases, expansion of place fields in CA1 (Miller and Best 1980; Brun et al. 2008; Kropff et al. 2015; Ormond and McNaughton 2015; Kanter et al. 2017). Conversely, inactivation of the HF disrupts MEC neurons’ ability to represent position and borders, while leaving the ability to represent head direction intact (Bonnevie et al. 2013). These findings indicate that HF and MEC mutually depend on each other to maintain accurate spatial representations.

The MEC, together with the lateral entorhinal cortex (LEC), gives rise to the largest cortical input to HF, and mediate the largest cortical output projection from HF (Lavenex and Amaral 2000; Nilssen et al. 2019). These projections from HF to EC originate in the subiculum (Sub) and CA1, and preferentially target MEC’s deep layers (Swanson and Kohler 1986; Kloosterman et al. 2003a, b; Witter 2006). Both are largely excitatory projections, mainly targeting spiny dendrites of principal neurons in layer V (van Haeften et al. 1995, Stewart 1997, Kloosterman, Van Haeften et al. 2003, Roth et al. 2016). Principal neurons in layer V of MEC in turn project to the neocortex (Köhler 1986; Swanson and Kohler 1986; Insausti et al. 1997) and to superficial layers II and III (Gloveli et al. 2001; van Haeften et al. 2003). Recently, it has been reported that these two output streams largely originate from two different populations of principal neurons. Whereas neurons in the superficial part (layer Va) seem to be the almost exclusive origin of projections to the neocortex (Sürmeli et al. 2015; Ohara et al. 2018), neurons in the deeper (layer Vb) give rise to projections targetting neurons in superficial layers II and III of MEC and in layer Va (Ohara et al. 2021). LV principal neurons are thus uniquely placed to relay hippocampally processed information to the neocortex as well as influencing neurons in the superficial layers of MEC that project to HF (Chrobak and Buzsaki 1994, Buzsáki 1996, Kloosterman, Van Haeften et al. 2003).

Layer V of MEC receives an additional input from the retrosplenial cortex (RSC; Jones and Witter 2007). In humans, RSC has been shown to be involved in autobiographical memory and planning (Vann et al. 2003), imagining fictitious experiences (Hassabis et al. 2007), and it is a part of the default mode network (Raichle et al. 2001). Furthermore, RSC has been suggested to contribute to spatial processing and memory and seems especially important for recognition of stable landmarks within an environment (Auger et al. 2012). Lesions to RSC result in navigational deficits in both humans and rodents (Valenstein et al. 1987; Maguire 2001; Haijima and Ichitani 2008; Pothuizen et al. 2008; Czajkowski et al. 2014) and a portion of RSC neurons show head direction cell activity (Cho and Sharp 2001; Jacob et al. 2017). In addition, the RSC has been found to code for the current path, position and turn direction within an environment, as well as processing spatial relationships between different subregions of complex paths (Alexander and Nitz 2015, 2017). There is also some evidence that RSC contains neurons with place-cell-like activity (Mao et al. 2017). Though our understanding of RSC’s function remains fragmented (Mitchell et al. 2018), we have some insight into the connectivity between RSC, PHR and the HF. RSC is extensively interconnected with Sub by way of excitatory reciprocal projections (Wyss and Van Groen 1992; Shibata 1994; Furtak et al. 2007, Sugar et al. 2011), whereas the connectivity of RSC with CA1 is sparse, mainly comprising some long-range inhibitory projections (Miyashita and Rockland 2007). Moreover, there is evidence that the reciprocal connections between RSC and Sub mature before eye‐opening and before the animal starts active exploration of the environment, and that these connections develop in parallel with the intrinsic connections of the HF and PHR (Haugland et al. 2019). Projections from RSC to MEC target dendritic spines and shafts of LV principal neurons (Czajkowski et al. 2013). The postsynaptic targets of RSC inputs are thus potentially similar to those of subicular projections. Although both RSC and subicular projections terminate throughout the deep layers of MEC, there is some evidence that subicular input might innervate principal neurons in both LVa and Vb, with a preference for neurons located in sublayer Vb (Köhler 1985; Kloosterman et al. 2003a, b; Sürmeli et al. 2015). Recent data has indicated that while both populations receive HF projections from CA1, the input to cells in LVb appears to be stronger (Rozov et al. 2020). Whether projections from RSC show a neuronal preference has not been addressed, though we know that many of the LV neurons receiving RSC projections send axon collaterals to superficial layers of MEC (Czajkowski et al. 2013). In view of recent findings that neurons in MEC layer Vb are the main origin of these superficial projections (Ohara et al. 2018), it thus seems plausible that the RSC and Sub target the same neurons in MEC, namely those that project superficially. However, this needs to be determined since it could still be that both input regions interact with segregated neuron populations within layer Vb of MEC. In the current paper, we investigated whether retrosplenial and subicular axons converge on single, superficially projecting LV neurons, using triple neuroanatomical tracing and confocal laser scanning microscopy. The present data clearly support the concept of convergence of subicular and retrosplenial inputs onto single-layer V superficially projecting neurons. We further conclude that synaptic contacts target the same parts of dendrites of those neurons, suggesting that integration of the two information channels might happen such that both inputs have a similar impact on the eventual firing of the neuron.

## Materials and methods

### Animals

We used 28 young adult female Sprague Dawley rats (Charles River) for this study. The weight of the animals ranged between 180 and 245 g. Animals were housed in a controlled environment (21 ± 1 °C, 40% humidity; lights off between 8:00 AM and 8:00 PM) in cages with 2–3 littermates before surgery and housed individually in the same environment post-surgery. Food and water were available ad libitum. The experimental protocols followed the European Communities Council Directive and the Norwegian Experiments on Animal Act and were approved by local and national authorities.

### Triple retrograde tracer injection

Animals were anesthetized with isoflurane and injected subcutaneously with atropine (Nycomed Pharma; 0.04 mg/kg), bipuvacain (Marcain, AstraZeneca; 1 mg/kg) and either buprenorphine (Temgesic, RB Pharmaceuticals; 0.05 mg/kg) or carprofen (Rimadyl, Pfizer; 5 mg/kg). After deep anesthesia was confirmed, the animals were mounted in a stereotactic frame and kept under isoflurane anesthesia throughout the surgery. Small holes were drilled in the skull bilaterally to uncover the midsagittal sinus, transverse sinuses and the three injection sites, and the dura was cut using the tip of a syringe needle. The stereotaxic coordinates for each injection were based on the Paxinos and Watson rat brain atlas (1998). The anterior edge of the transverse sinus was used as a reference point for the anteroposterior (AP) coordinates ipsilaterally, the midsagittal sinus for the mediolateral (ML) coordinates and the cortical surface for the dorsoventral (DV) coordinates (depth). For injections into the superficial layers of MEC, a 1 µl Hamilton syringe was filled with the retrograde tracer Fast Blue (FB, EMS-Chemie; 1% solution in 0.1 M phosphate-buffered saline (PBS), pH 7.4), and lowered into the brain (AP 1.3 mm; ML 4.6 mm; DV 4.8 mm), where we injected 150 nl mechanically over 5 min. For the injection of anterograde tracers into subiculum and RSC, we used a 5% solution of biotinylated dextran amine (BDA; 10,000 MW, Invitrogen, #D1956) and 2.5% *Phaseolus vulgaris-*leucoagglutinin (PHA-L; Vector Laboratories, #L-1110), respectively. Glass micropipettes (GCI120F-10, Harvard Apparatus) with an inner tip diameter 9–15 µm were filled with the appropriate tracer and lowered into stereotaxic position. The tracers were injected iontophoretically using a positive pulsed direct current (current 6 µA and 7.5 µA for BDA and PHA-L, respectively; 6 s on, 6 s off) for 10 min. We injected anterograde tracers at three dorsoventral levels at 0.3 mm intervals (RSC coordinates: AP 2.1 mm; ML 1.0 mm; DV 1.2, 1.5, 1.8 mm; Subiculum coordinates: AP 2.35 mm; ML 5.2 mm; DV 4.2, 4.5, 4.8 mm). For all tracer injections, we waited 5 min prior to and after the injection, before retracting the syringe or capillary slowly from the brain. Finally, we sutured the skin over the wound and the animal could recover. Subcutaneous injections of either buprenorphine or carprofen were given as pain relief for 24 h after surgery.

Some animals (*n* = 9) were injected unilaterally with all three tracers in either the left (*n* = 2) or the right (*n* = 7) brain hemisphere. In three of the cases reported, BDA was injected in the RSC and PHA-L in the subiculum, and injections were placed in both brain hemispheres. In the remaining animals, BDA was injected in the subiculum and PHA-L into the RSC.

### Perfusion, evaluation of injections and intracellular injection

After a survival period of 10–14 days, the animals were sacrificed with an intraperitoneal overdose of Equithesin (i.p. 11 mg/kg bodyweight; Sanofi Sante). Next, they were transcardially perfused with 250 ml of oxygenated Ringer solution (145 mM NaCl, 3 mM KCl, 2 mM NaHCO_3_, pH 6.9, 37 °C) immediately followed by 250 ml iced, 4% freshly depolymerized and filtered paraformaldehyde (Merck) in 0.125 M phosphate buffer (PB). The brain was removed and post-fixed in paraformaldehyde for 2 h, before being rinsed with PB and stored 1–7 days in PB at 4 °C. Horizontal slices were cut with a vibratome (Leica VT1000S; bath fluid 0.125 M PB). Cutting of 100 µm thick sections started at the most dorsal level of the brain hemisphere, while alternating 100 µm/400 µm slices were obtained after reaching the most dorsal part of MEC. 400 µm slices were stored 3–7 days in PB at 4 °C.

The 100 µm sections were permeabilized 3 × 10 min in 0.125 M PB with 1% Triton X-100 (Merck; pH 7.4, the joint solution hereby referred to as PB-Tx) and preincubated with 5% donkey serum (Sigma, #D9663) in PB-Tx for 3 h. Subsequently, we incubated these sections with goat anti-PHA-L (1:1000; Vector Laboratories, #AS-2224, RRID: AB_2315136) in 5% donkey serum PB-Tx for 48 h at 4 °C and then in AlexaFluor-488-conjugated streptavidin (1:200; Invitrogen, #S32354, RRID: AB_2315383) and secondary antibody, AlexaFluor-546-conjugated donkey anti goat (1:400; Invitrogen, #A-11056, RRID:AB_142628) in 5% donkey serum PB-Tx for 2 h. The samples were then washed in PB 3 × 10 min, suspended in 0.1 M Tris(hydroxymethyl)-aminomethane-HCl (Tris–HCl, pH 7.6; 252,859, Merck) and mounted on superfrost slides (Thermo Scientific).

We imaged the 100 µm sections using a fluorescent scanner (Carl Zeiss, Jena, Germany, Mirax Midi BF/FL v 1.12) equipped with an Axiocam digital camera (Carl Zeiss, Jena, Germany). Filters sets with excitation filter/beam splitter/emission filter Ex. G365/BS FT395/Em. LP 420, Ex. BP470/40/BS FT495/Em. BP525/50, and Ex. BP545/25/BS FT 570/Em. BP605/70 (All Carl Zeiss, Jena, Germany) were used to image FB, AlexaFluor 488 and AlexaFluor 546 (AF488 and AF546), respectively. The images were used to confirm the correct placement of tracer injections and to ascertain overlap between BDA-positive axonal terminals, PHA-L-positive axonal terminals and retrogradely FB-labeled neurons in deep layers of MEC. 400 μm thick sections adjacent to the 100 μm sections with a confirmed overlap of all three labels present were used for intracellular injections.

Suitable 400 μm slices were washed in PB and mounted in a PB-filled chamber on a fixed-stage upright fluorescent microscope (Axio Examiner, Zeiss, Germany) equipped with a PlanApo 20X 1.0 NA water dipping lens, a micromanipulator (Luigs Neuman, Germany) and an iontophoretic injection device capable of delivering nA-currents (custom made appliance). The sections were imaged with IR-DIC to resolve cell details and with a fluorescent filter set 75HE (Zeiss, Ex. G365, BS FT 395, Em. BP 445/50, separated from the IR-DIC by FT 660) to visualize FB together with Alexa Fluor 568 (AF568). We randomly selected FB-labeled cells in deep layers of MEC and filled them intracellularly by impaling cell bodies with a 70–130 MΩ glass pipette (GCI120F-10, Harvard Apparatus; microelectrode-tip diameter 0.5–0.8 μm) containing AF568 (Invitrogen, #A-10441; 10 mM in 200 mM KCl). Negative current was applied to the micropipette (2 nA, 500 μs on; 500 μs off), for at least 10 min to completely fill the impaled cell and its dendrites. We judged filling as complete when the finest details, such as dendritic spines and thin distal branches of dendrites far away from the cell body, became visible. After filling, the sections were transferred to 4% paraformaldehyde and postfixed overnight at 4 °C. Next, the sections were rinsed 3 × 10 min in PB, permeabilized 7 × 10 min in PB-Tx and preincubated in PB-Tx with 5% donkey serum for 3 h, followed by an incubation with goat anti-PHA-L antibody (1:1000) in 5% donkey serum PB-Tx for 72 h at 4 °C. Subsequently, the slices were rinsed 7 × 10 min in PB-Tx and stained with AF488-conjugated streptavidin (1:200) and AF633-conjugated donkey anti-goat antibody (1:400; Invitrogen, #A-21082, RRID:AB_141493) in 5% donkey serum PB-Tx overnight in room temperature.

Sections were washed in PB-Tx 3 × 10 min each, dehydrated in ascending ethanol series (30%, 50%, 70%, 90%, 100%, 10 min each), permeabilized in a 1:1 mixture of 100% ethanol and methyl salicylate (Sigma Aldrich) for 10 min, then cleared and mounted in methyl salicylate. Slides were stored at 4 °C in methyl salicylate. Confocal image stacks were taken from areas of interest where dendritic branching of the intracellularly filled cell overlapped both RSC and subicular axonal plexuses (see below).

### Staining for synaptic markers

In some cases where BDA had been injected into subiculum (*n* = 5), we stained 400 µm sections with AF488-conjugated streptavidin (1:200) prior to dehydration. Cases with well-developed subicular axonal plexus and satisfactory intracellular filling of FB positive LV neurons in MEC were chosen (*n* = 4). Following antibody staining, these sections were processed the same way as other 400 µm samples. After confocal imaging and 3D-reconstruction (see below), 400 µm sections were transferred to a 1:1 mixture of 100% ethanol and methyl salicylate for 10 min, rehydrated in a descending ethanol series (100%, 90%, 70%, 50%, 30%, 10 min each) and incubated in cryoprotective solution (30% sucrose in PB) overnight. Next, the samples were mounted in frozen 30% sucrose on a freezing microtome and re-sectioned into 50 µm thick sections. Sections were washed with PB 3 × 10 min, then 7 × 10 min in PB-T at room temperature and preincubated with PB containing 5% normal goat serum (NGS; DAKO, X0907) in 0.5% Triton-x-100 PB (PB-Tx 0.5%) for 2–3 h. Sections were subsequently incubated with polyclonal rabbit anti-synaptophysin antibody (1:100; Abcam, #ab14692, RRID:AB_301417) and a monoclonal mouse anti-PSD95 antibody (1:150, NeuroMab, #75–028, RRID:AB_2292909) in 5% NGS PB-Tx 0.5% for 72 h at 4 °C. Next, sections were washed 7 × 10 min in PB at room temperature and subsequently incubated for 24 h in a mixture of AF514-conjugated goat anti-rabbit antibody (#A-31558, RRID:AB_10375589), AF633-conjugated goat anti-mouse antibody (#A-21052, RRID:AB_2535719) and AF488-conjugated streptavidin (all 1:350; all Invitrogen). Lastly, preparations were washed in PB 3 × 10 min, suspended in 0.1 M Tris–HCl and mounted on superfrost slides.

### Staining of Ctip2 and Nissl substance

Some sections were selected for staining of Ctip2, a transcription factor shown to be a marker for neurons in deep LV of MEC (LVb, see Sürmeli et al. 2015). The 400 µm thick samples were dehydrated and resectioned into 50 µm sections as described above. Sections unlikely to contain the previously imaged cell bodies were suspended in 0.1 M Tris–HCl, mounted on Superfrost plus-slides, dried overnight and stained with cresyl violet for cytoarchitectonic orientation. The remaining sections were placed in 10 mM sodium citrate tribasic dihydrate (pH 8.5) and heated to 80 °C for 25 min. After cooling to room temperature, the sections were permeabilized by washing them 3 × 5 min in sodium citrate buffer containing 0,3% Triton-X-100 and subsequently preincubated in sodium citrate buffer with 0.3% Triton-X-100 and 3% bovine serum albumin (BSA; Sigma) for one hour. Next, monoclonal rat anti-Ctip2 (1:3000; Abcam, #ab18465, RRID:AB_2064130) was added to the solution and the sections were left on a shaker in room temperature overnight. Next, the samples were washed 2 × 15 min in a solution with 10 mM citrate buffer, 0,1% Triton-X-100 and 1% BSA before polyclonal biotin-conjugated rabbit anti-rat IgG (1:400, Vector labs, #BA-4001, RRID:AB_10015300) was added to the solution. After 90 min the sections were washed 3 × 5 min in Tris–HCl before the Ctip2-antibody complexes were visualized using 3,3’-Diaminobenzidine tetrahydrochloride (DAB, Sigma-Aldrich) as the chromogen. Sections were suspended in Tris–HCl and mounted on Superfrost slides before coverslipping.

### Laser scanning microscopy

Sections were imaged in sequential scanning mode with a Zeiss Meta 510 confocal instrument (Carl Zeiss, Jena, Germany) using Plan-Apochromat 10X0.45 NA air, 20X/0.8 NA air and 63X/1.4 NA Oil DIC objectives corrected for both chromatic and spherical aberration. Images of BDA-labeled fibers stained with streptavidin conjugated with AF488, AF568 and AF633 obtained with the same scanning parameters as used for the experimental sessions, were used to determine whether and to what degree correction was necessary to compensate for possible errors in standard scanning parameters (Wouterlood et al. 2003).

FB was excited by a diode laser emitting 405 nm (BS 405 nm, Em. 420–480 nm), A488 was excited by an Argon laser (BS 488 nm, Em. 505–550 nm), A568 was excited by a Helium-Neon laser (BS 561 nm, Em. 575–615 IR) and A633 was excited by a Helium-Neon laser (BS 633, Em. LP655). For triple imaging, we used a combined filter set (HFT 405/488/561/633/KP725).

Sections were first scanned at 10X, resolution of 1024 × 1024, 8-bit sampling, and z-increment of 5 μm. Intracellularly injected neurons were localized and we verified whether these cells were situated in deep layers of MEC where BDA- and PHA-L-labeled fibers were present as well. Next, cell bodies of the filled cells were double scanned with diode 405 nm and He/Ne 561 nm laser lines at 20X, resolution 1024 × 1024, 8-bit depth, z-increment of 1 μm, to confirm that the intracellularly AF568-filled cells were indeed retrogradely FB-labeled. For high-resolution 3D analysis, samples were scanned in all channels with the high-resolution 63X immersion objective lens, 1024 × 1024 pixels in the x–y axes, 8-bit sampling, *z*-increment 0.35 µm. The optical section thickness was set to 0.7 µm by adjusting the pinhole separately for each channel. After 3D-reconstruction, putative contacts were imaged in detail using the same objective at a resolution of 256 × 256 pixels, zoom 6, 8-bit sampling and z-increment 0.35 µm.

### Imaging of synaptic markers

Assessment of quadruple staining of synaptic contacts and spectral unmixing of closely located fluorescence peaks was performed using a Zeiss LSM 780 microscope (Carl Zeiss, Jena, Germany) with ZEN software. For each of the fluorochromes (AF488, AF514, AF568 and AF633) we acquired a reference emission spectrum using simultaneous excitation with Argon laser and DPSS diode (λ_ex_ = 488 nm and 561 nm, respectively, BS 488/561/633, λ_em_ = 499–691 nm, *Δ*λ = 8.7 nm, while excluding the 8.7 nm channel spanning the 561 laser line), over a Plan-Apochromat 63X/1.40 NA Oil DIC objective lens at a resolution of 348 × 348 pixels, zoom 6, 8-bit sampling and the z-increment 0.35 µm. The same singly labeled samples were also used to calibrate acquisition parameters. Quadruple-labeled samples were subsequently imaged using the same settings. Linear unmixing with the Zen software was then performed to visualize each channel.

### Imaging of Ctip2-positive neurons

Samples immunostained for Ctip2 and visualized with DAB, as well as adjacent sections stained with cresyl violet, were imaged using an Axio Scan.Z1 slide scanner with a Plan-Apochromat 40X/0.95 NA Ph 3 M27 objective in brightfield mode. Sections containing AF568-filled neurons were imaged using a 20X/0.8 NA M27 objective with white LED illumination (Ex. 533–558 nm, BS 560 nm, Em. 570–650 nm). We adjusted the brightness and contrast of brightfield and fluorescent images using Adobe Photoshop CC 2017 and used Adobe Illustrator CC 2017 to delineate brain areas and to assess the overlap between AF568-filled and Ctip2-positive neurons.

### 3D reconstruction and mapping

Dendritic reconstruction was conducted as described previously (Kononenko and Witter 2012). We used a custom plugin, Skeleton tool (Schmitt et al. 2004; Evers et al. 2005), for Amira 5.3.3 to trace the dendritic trees and positions of the spines. For the Sholl analysis (Sholl 1953), the number of branches crossing concentric spheres of increasing radius (*Δ*r = 10 µm) originating at the soma was counted. Spine density was expressed as the number of spines per 10 μm of dendrite length. To quantify the number of BDA- and PHA-L-filled putative axonal boutons and their distribution along reconstructed dendrites, the dendritic surface was expressed as triangles (Evers et al. 2005). The BDA- and PHA-L-positive pixels were identified by thresholding the AF488 and AF633 channels after the Otsu method (Otsu 1979), which seeks to set a threshold level that clusters the intensities of pixels in our scanned image such that we minimize the overlapping signal between separate objects. The proximity of BDA (AF633) and PHA-L-positive (AF488) voxels within 300 nm from each surface element identified as a triangle was calculated and expressed as a heat map on the dendritic surface. A separate heat map was calculated for each of the tracers. Finally, for each putative contact, the zoomed image was analyzed and it was determined whether it conformed to previously established criteria of a presynaptic bouton (Wouterlood et al. 2008), being an axonal swelling having a diameter 3 times bigger than the preceding and continuing fiber.

### Synaptic reconstruction

Images of quadruple-labeled samples (presynaptic fibers, AF488; synaptophysin, AF514; postsynaptic dendrites, AF568; postsynaptic PSD-95, AF633) were loaded into ZEN software, and previously identified putative synaptic contacts were located using the AF488 and AF568 channels. The distribution of each fluorochrome along the axis connecting the center of a presynaptic bouton and the center of the dendritic spine (for spine synapses) or dendritic segment (for shaft synapses) was measured. Putative contacts showing local labeling peaks for both synaptophysin (AF514) and PSD-95 (AF633) along that axis (located between peaks for AF488 and AF568) were considered synaptic. Given the reduced Z-axis resolution in optical microscopy, only synapses found in the x–y plane were measured. 3D reconstructions of the synapses were performed in Amira version 5.3.3.

### Morphological and statistical analysis

For statistical analyses, morphological parameters from ASCII-tables generated in Amira were imported into Excel and MatLab. Lengths of dendrites and distances from the soma to each putative synapse were calculated by summation of dendritic segments between the soma and each contact. Dendritic spines were similarly located by distance from the soma and binned in successive 20 µm bins along the dendrites. Means were calculated and variance was expressed as standard deviations. The cumulative distribution of putative synaptic contacts onto dendritic spines and dendritic shafts were represented separately for both retrosplenial and subicular inputs and were compared using a Kolmogorov-Smirnoff test. The total percentage of BDA-positive pixels within each image stack was used to estimate the density of the labeled axons. The correlation between the density of plexus and the frequency of synaptic contacts assessed in the confocal microscope was tested with *F* test for linear regression. The distribution of contact frequencies normalized to the plexus density was tested with D’Agostino-Pearson normality test (“omnibus K2” version). Significant effects were accepted at *p* < 0.05.

## Results

### Morphology of superficially projecting LV MEC neurons

**Fig. 1:**
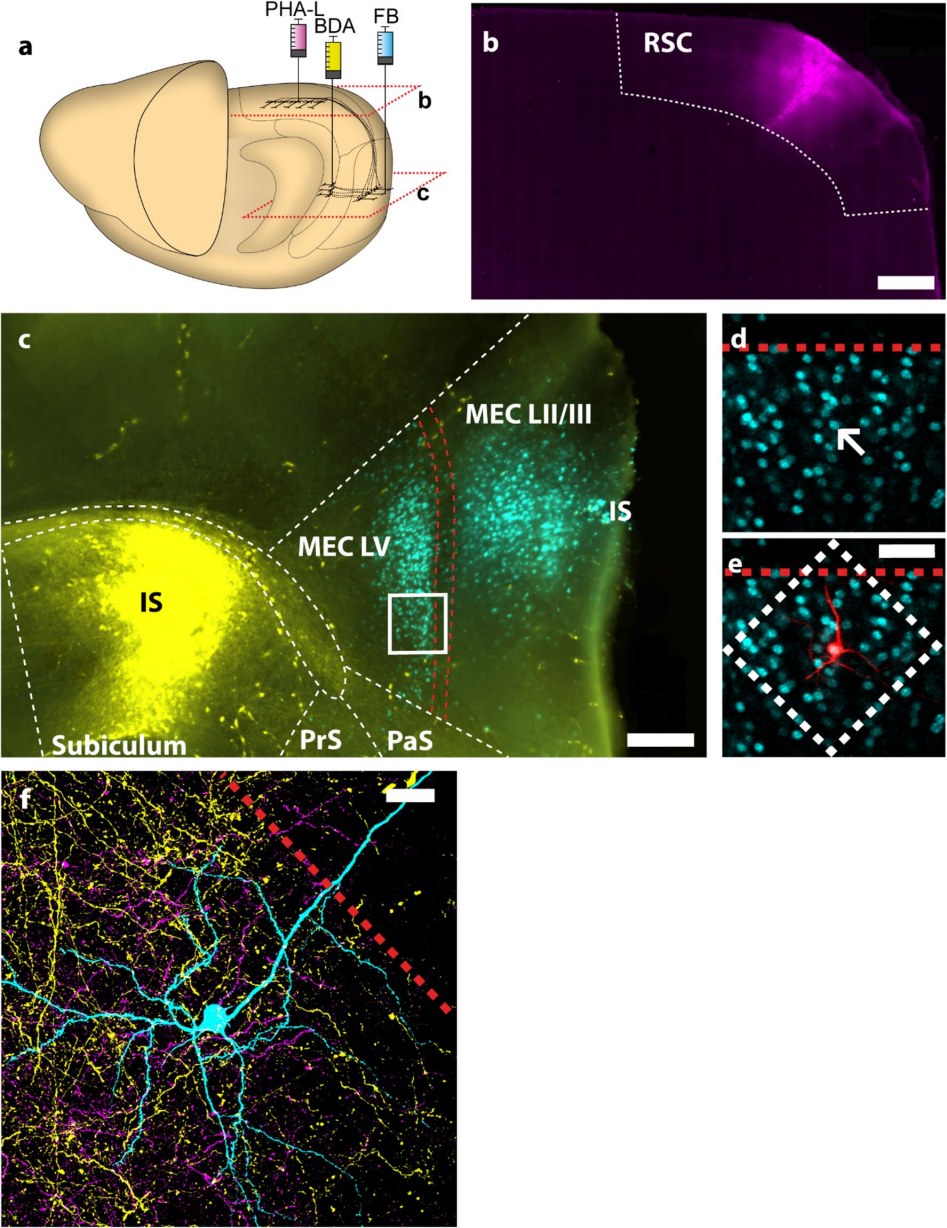
Experimental design. **a** Schematic illustration of a rat brain and the injection sites. FB (cyan) was injected into the superficial layers of MEC. One of the anterograde tracers BDA (yellow) or PHA-L (magenta) was injected into the RSC, whilst the other was injected into the subiculum. **b** and **c** indicate the levels of panels **b** and **c**. **b** PHA-L (magenta) injection site in RSC. Scale bar 1000 µm. (**c**) BDA injection site in subiculum (IS; yellow) and most ventral tip of FB injection site (IS) in superficial MEC. Red lines indicate lamina dissecans. Sections with a thickness of 100 µm were used to select 400 µm thick sections suitable for intracellular injection. The white rectangle indicates the zoomed region of the corresponding area in an adjacent 400 µm section depicted in **d** and **e**. Scale bar 500 µm. **d** FB-positive neurons in layer V of MEC. Target neuron for intracellular filling indicated (arrow). Lamina dissecans border indicated with a red line. **e** Filled neuron (red) overlapping with FB. White rectangle indicates the XY-plane limits of the confocal image stack maximum projection shown in **f**. Scale bar 20 µm. **f** Maximum projection image of confocal stack depicting filled neuron (cyan), BDA-positive axonal fibers (yellow) and PHA-L-positive axonal fibers (magenta). Scale bar 20 µm. 1a adapted from (Czajkowski et al. 2013)

**Fig. 2:**
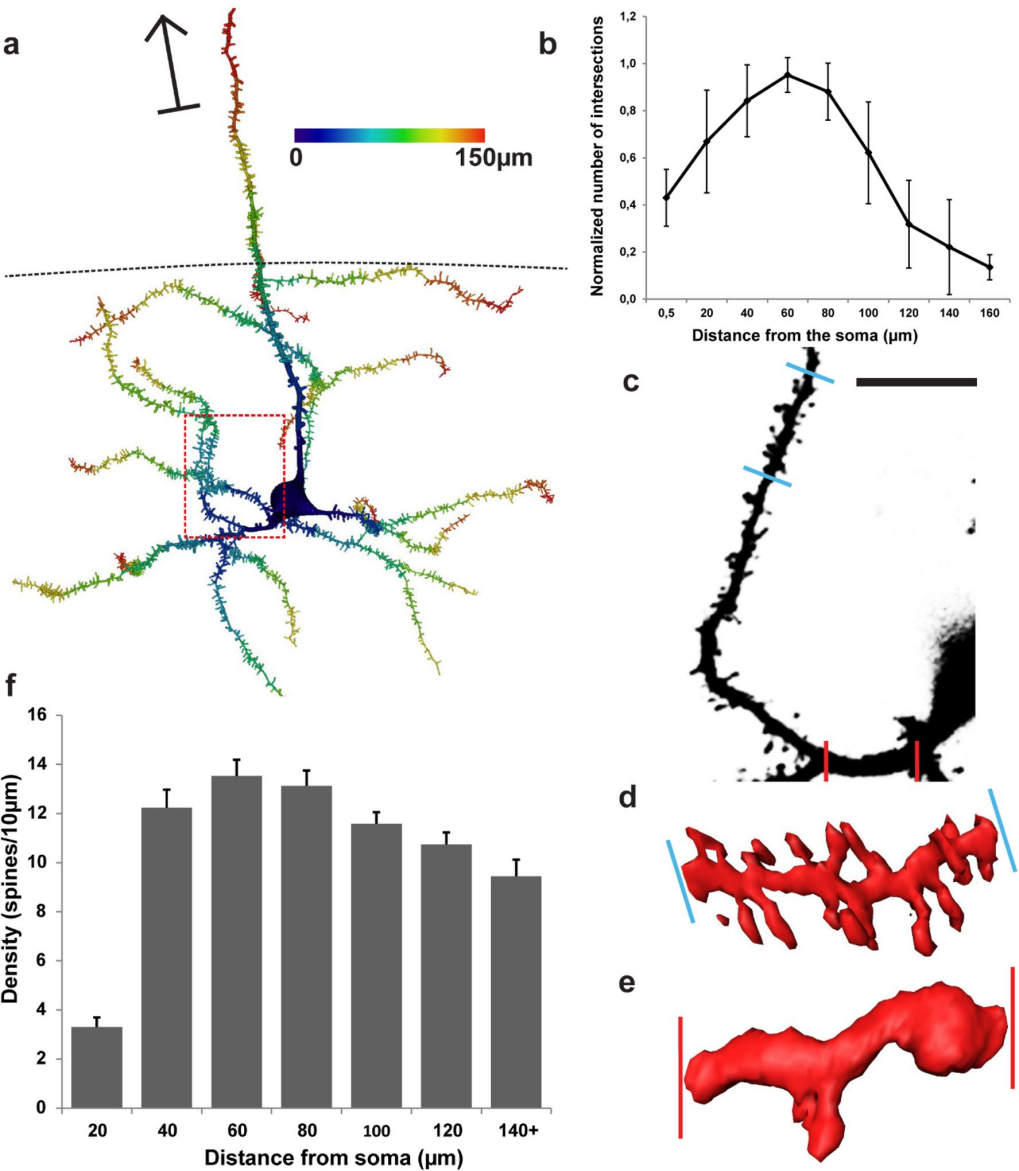
Morphological properties of MEC LV neurons. **a** A representative example of a reconstructed LV principal neuron based on the image stack in Fig. 1f. Distance from soma is represented by blue to red coloring. Arrow points toward the pia. Dashed line indicates the border between LVa and LVb. Red dashed box indicates area depicted in panel **c**. **b** Sholl analysis illustrating the change of dendritic complexity in relation to distance from the soma (*n* = 27 from 8 animals). Sholl values were normalized to the number of first-order dendrites. Error bars show SEM. **c–e** Comparison of spine density between proximal and distal parts of the dendritic tree. **c** High-resolution representation of the dendrite taken from the boxed area in **a**, showing the selected proximal (red bars) and distal (blue bars) parts of the dendrite. Scale bar 10 µm. **d** Example of the distal part (60–70 µm from the soma). **e** Example of the proximal part (0–10 µm) from the soma). **f** Spine density along the length of the dendritic tree as measured by distance from the soma. Bin size 20 µm. Error bars show SEM

We confirmed that injection sites were confined to the target areas (Fig. 1) and verified the overlap between retrogradely FB-positive LV MEC neurons and BDA- and PHA-L-positive axons from the subiculum and retrosplenial cortex (Fig. 1b, c). We had successful triple injections in 7 animals. A total of 63 randomly selected FB-positive LV MEC neurons within the two overlapping axonal plexuses were filled with A568 (Fig. 1d and e). Of these, 27 neurons were judged to be sufficiently filled and were selected for confocal imaging (Fig. 1f). 3D reconstructions of the imaged neurons were made using Amira software with custom plug-ins ( Schmitt et al. 2004; Evers et al. 2005) like we have described before (Kononenko and Witter 2012; Czajkowski et al. 2013). All reconstructed neurons were classified as principal neurons, showing dendritic spines and dendritic morphology in line with previous descriptions (Hamam et al. 2000; Canto and Witter 2012). Following the terminology of the latter study, we identified 2 multipolar neurons, 3 horizontal neurons and 22 multidirectional pyramidal neurons. Multipolar neurons possessed no primary apical dendrite and had many dendrites radiating from all over the soma, though they were largely confined to the deeper layers. Both horizontal and multidirectional pyramidal cells had round to pyramidal somata and a thick, spiny apical dendrite extending superficially toward the pia. Horizontal pyramidal neurons were distinguished from multidirectional pyramidal cells by a basal dendritic tree which mainly extended in parallel to the lamina dissecans. In contrast, multipolar pyramidal neurons showed basal dendrites that coursed horizontally, vertically, and obliquely in LV and sometimes extended into LIII and LVI. Horizontal and multidirectional pyramidal neurons displayed no differences in other properties. A representative multidirectional pyramidal cell is shown in Fig. 2a, as it represents the most abundant morphological type we encountered in this study. The apical dendrite often bifurcated deep to the lamina dissecans, with second and third-order dendrites sometimes extending through the lamina dissecans into LIII.

All 27 cells were pooled together in the following analyses. Since our primary objective was to investigate possible convergent input to principal LV neurons, and projections from both RSC and Sub show a strong preference for LV (Köhler 1985, Kloosterman et al. 2003a, b, Sürmeli, Czajkowski et al. 2013), the following analyses were focused on dendrites contained within LV.

A typical principal neuron displayed between 3 and 9 primary basal dendrites arising from the soma and extending for up to 200 µm. We conducted Sholl analysis to assess dendritic complexity by counting the number of dendritic intersections with a Sholl sphere as a function of distance from the soma. Dendritic complexity peaked around 60 µm distance from the soma (Fig. 2b). Proximal basal dendrites were sparsely spiny, showing approximately 3 spines/10 µm for the first 20 µm. The number of spines increased rapidly over the next 20 µm, often after the first dendritic bifurcation, and eventually peaked at 60 µm distance with a density of 13 spines/10 µm (Fig. 2c–f).

### Putative contacts from RSC and subiculum converge onto single neurons

**Fig. 3:**
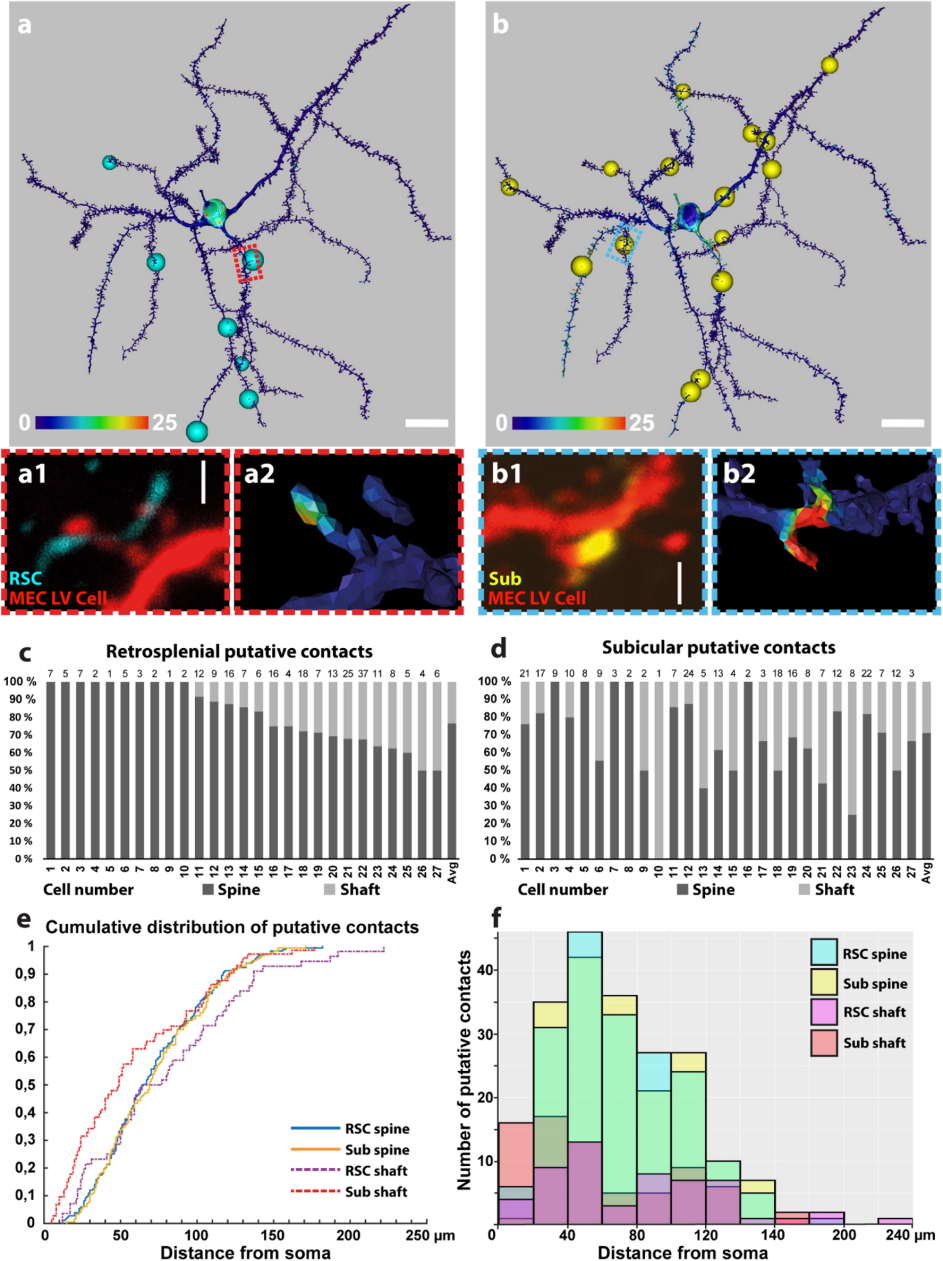
Convergence of putative RSC and Sub contacts onto single MEC LV neurons. **a** Surface rendering of RSC input mapping of the cell imaged in Fig. 1f and depicted in Fig. 2a (cell number 19). Staining intensity of anterograde tracer injected into RSC (in this case PHA-L) within 300 nm of the reconstructed surface shown color-coded as a heat map. Cyan spheres indicate points fulfilling the criteria for a putative synaptic contact. Red rectangular box indicates the region shown in **a1–a2**. Scale bar 20 µm. **a1** Close-up example of a putative contact between PHAL-positive axonal bouton and a spine. Scale bar 1 µm. **a2** Heat map surface rendering showing the proximity of PHA-L-positive axon and the reconstructed cell surface. **b** Surface rendering of subicular input mapping onto the same neuron (#19) depicted in **a**. Staining intensity of anterograde tracer injected into subiculum (in this case BDA) within 300 nm of the reconstructed surface shown color-coded as a heat map. Yellow spheres indicate points fulfilling the criteria for a putative synaptic contact. Cyan rectangular box indicates the region shown in **b1**–**b2**. **b1** Close-up example of a putative contact between BDA-positive axonal bouton and a spine. Scale bar 1 µm. **b2** Heat map surface rendering depicting the proximity of BDA-positive bouton and the reconstructed cell surface. **c** Bar graph showing the proportion of putative spine and shaft contacts with axonal fibers stemming from anterograde tracer injection in RSC (Y-axis) for all individual reconstructed neurons (1–27) and the average proportion (X-axis). Absolute number of contacts displayed above each bar. (**d**) Bar graph showing the proportion of putative spine and shaft contacts with axonal fibers stemming from anterograde tracer injection in subiculum (Y-axis) for all individual reconstructed neurons (1–27) and the average number (X-axis). Absolute number of contacts displayed above each bar. **e** Cumulative fraction of putative contacts along the dendritic length of reconstructed neurons. RSC spine contacts represented in cyan, subicular spine contacts in yellow, RSC shaft contacts in dashed purple and subicular shaft contacts in dashed red lines. **f** Histogram showing number of putative synaptic contacts (Y-axis) along the dendritic length (X-axis). RSC spine contacts represented in cyan, subicular spine contacts in yellow, RSC shaft contacts in purple and subicular shaft contacts in red columns. Green colour is a result of histograms representing RSC and subicular spine contacts overlapping. Bin size 20 µm

The 3D-reconstruction of filled neurons allowed us to map the proximity of BDA-positive and PHA-L-positive axonal fibers onto the rendered dendritic surface (Schmitt et al. 2004; Evers et al. 2005). Labeled axons within one voxel (300 nm) proximity to the surface were expressed as a heat map relative to the fluorescence intensity of the axonal fibers. Separate heat maps were created for each of the two fluorescently labeled anterograde tracers BDA and PHA-L (Fig. 3a, b). Hotspots representing proximity between putative presynaptic and postsynaptic labels (Fig. 3a and b1) were examined to verify the presence of presynaptic boutons. Axonal swellings were classified as boutons if the diameter of the swelling was at least three times bigger than the preceding and continuing fiber (Wouterlood et al. 2008).

All 27 cells in the data set presented here displayed putative synaptic contacts with both BDA and PHA-L positive fibers. An example cell is shown in Fig. 3a, b, where BDA and PHA-L positive fibers contact the same neuron. The remaining 26 neurons with locations of putative contacts are shown in supplementary Fig. 1. The total number of retrosplenial and subicular putative contacts for each individual cell is shown in Fig. 3c and d, respectively. Out of the population of retrosplenial putative presynaptic terminals, 77% (183/239) contacted dendritic spines, whilst the remaining 23% (56/239) contacted dendritic shafts (Fig. 3c). Out of the population of subicular putative presynaptic terminals, 71% (180/253) contacted spines while the remaining 29% (73/253) were on dendritic shafts (Fig. 3d).

The distribution of contacts along the distance of the dendritic tree was plotted for the pooled retrosplenial and subicular putative contacts (Fig. 3e and f). Subicular and retrosplenial putative contacts with spines typically intermingled on the proximal part of the apical dendrite and on several of the basal dendrites. The distribution for spine contacts was remarkably similar (Kolmogorov–Smirnov test *p* = 0984), with contacts from both inputs overlapping on the same portions of the dendritic tree, though subicular shaft contacts were positioned closer to the soma (Kolmogorov–Smirnov test *p* = 0.033). It is of interest to note that in some instances two contacts with a different origin showed close proximity, less than 10 µm (supplementary Fig. 2), but they could also be further apart. We did not quantify this since we could not control for the overall density of labeling of the two inputs. However, we did verify whether the density of putative subicular contacts on the dendrites of each MEC layer V cell followed the Peters' rule (Peters and Payne 1993), as we did for RSC inputs in our previous paper (Czajkowski et al. 2013). For all pyramidal neurons, the frequency of detected contacts was proportional to the density of incoming fibers surrounding the dendritic tree. The correlation was linear (*R*^2^ = 0.78), and the distribution of contact frequencies normalized to the plexus density was unimodal (*p* > 0.1). This suggests that the entire population of MEC neurons is innervated equally by the subicular projections.

### The distributions of pre- and postsynaptic proteins indicate the presence of synaptic contacts

**Fig. 4:**
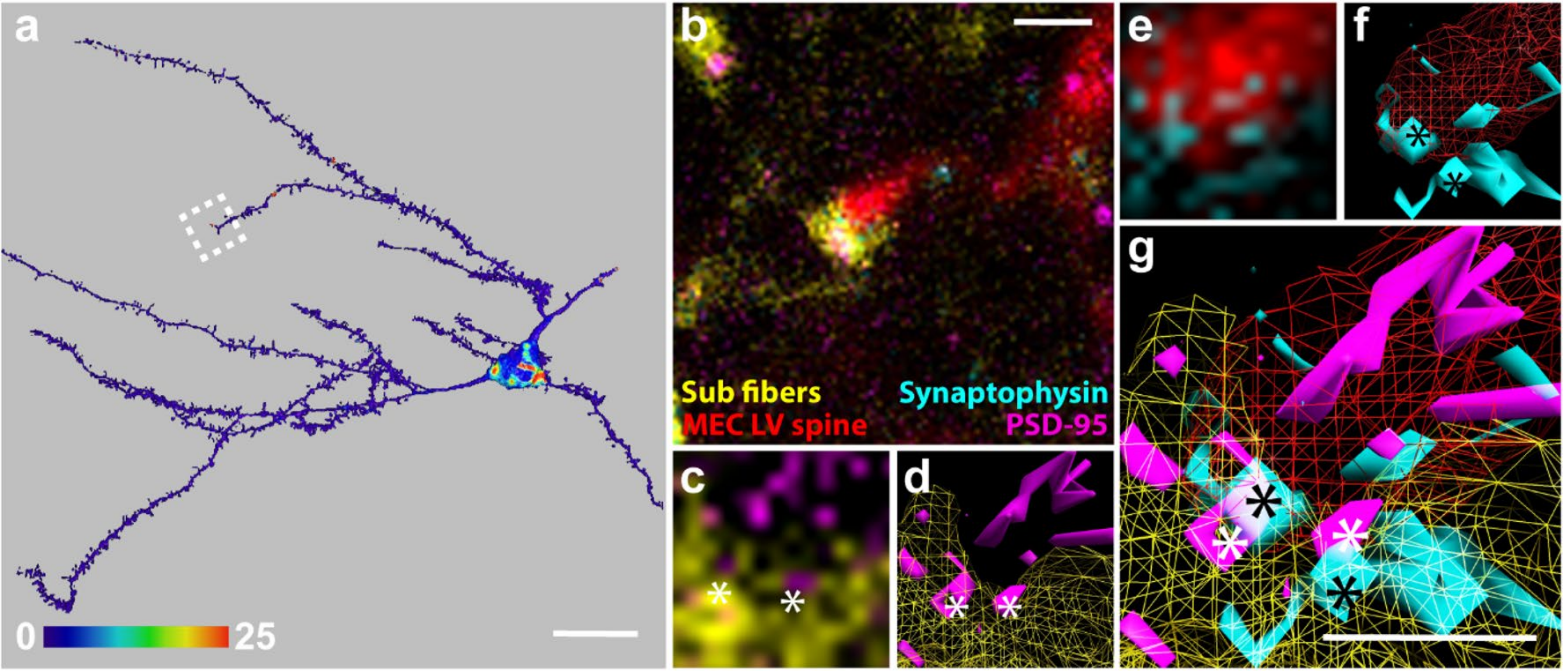
Staining of subicular putative contacts for synaptic proteins synaptophysin and PSD-95. **a** Surface rendering of a reconstructed neuron. Staining intensity of anterograde tracer BDA, injected in the subiculum, within 300 nm of the reconstructed surface is shown color-coded as a heat map. Dashed white square shows the area depicted in **b**. Scale bar 20 µm. **b** One plane of a confocal image stack showing an Alexa 568-filled dendritic spine (red), BDA-positive axonal fibres with boutons (yellow), labelling for PSD-95 (magenta) and synaptophysin (cyan). Scale bar 1 µm. **c** Overlap between BDA-positive synaptic bouton and PSD-95. **d** 3D-reconstruction of the image in **c.** White asterisks show overlapping elements. **e** Overlap between Alexa-568 filled dendritic spine and synaptophysin. **f** 3D-reconstruction of the image shown in **e**. Black asterisks show overlapping elements. **g** Reconstructions **d** and **f** merged, showing two points of overlap where overlapping elements of PSD-95 and synaptophysin are located between an Alexa-568-filled dendritic spine and a BDA-positive synaptic bouton, which also show some degree of overlap. Scale bar 0.5 µm

Four additional well-filled neurons embedded in densely labeled axonal plexuses were selected for synaptic staining of subicular putative contacts. We immunolabeled for synaptophysin and PSD-95 as pre- and postsynaptic markers respectively, to assess the presence of synapses onto these four neurons. 51 putative contacts were examined of which 43 (84%) were spine contacts and 8 (16%) were contacts on dendritic shafts. Of these, 7 (16% of total) spine contacts and 2 (25% of total) shaft contacts were positive for both synaptophysin and PSD-95. In these cases, the synaptic markers overlapped both with each other and with boutons and the filled elements of the neurons, respectively (Fig. 4a–g; see also Czajkowski et al. 2013 for a similar procedure used to assess the presence of retrosplenial putative contacts). This indicated that in the most conservative possible assesment about 17% of the total putative contacts were indeed synapses. Given the fact that both synaptophysin and PSD-95 are not necessarily present at all synapses, this number is likely an underestimate. Apart from contacts containing both markers, we also detected 19 contacts with only synaptophysin labeling (37%) and one contact with just PSD-95 presence (2%).

### Superficially projecting cells are mainly LVb pyramidal neurons.

**Fig. 5:**
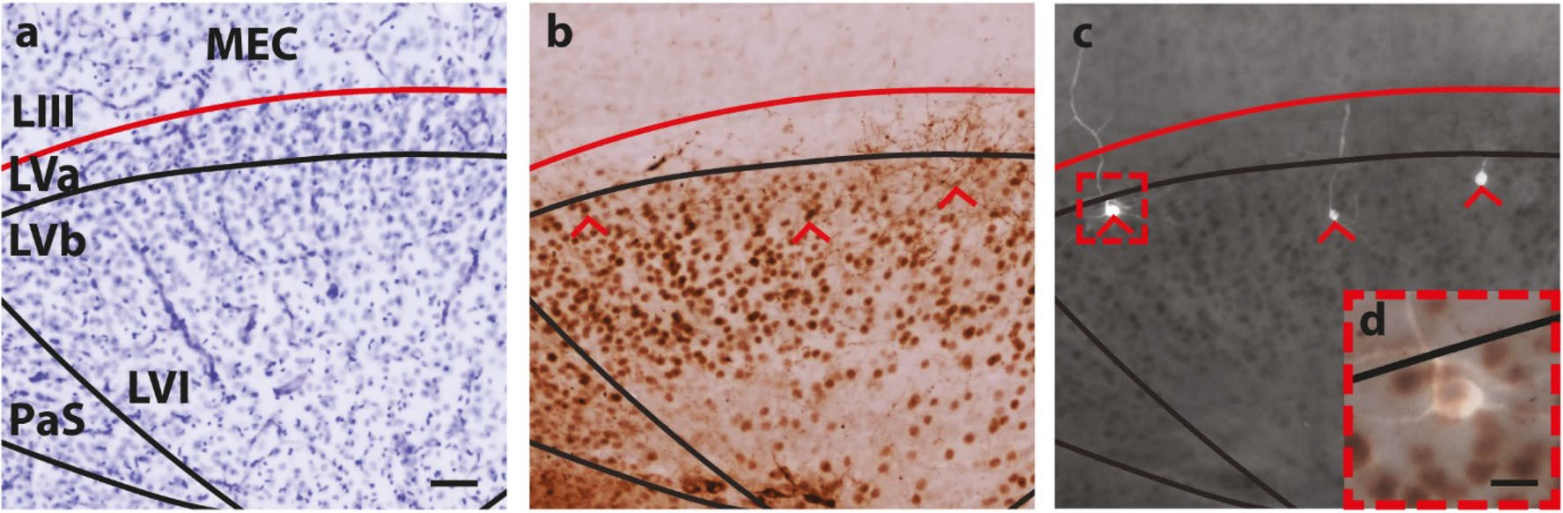
Staining for Ctip2. **a** Cresyl violet stain and delineation of deep regions of MEC and adjacent areas, including parasubiculum (PaS). Red line shows the border between the lamina dissecans and LV. Scale bar 50 µm. **b** Ctip2-positive neurons forming a thick band of neurons in LVb, while LVa contains only a few, weakly positive neurons. Axonal fibers are seen as a result of DAB staining of BDA injection in subiculum. Red arrowheads indicate locations of AF568-filled neurons in **c**. The rightmost neuron is Ctip2 negative, whilst the other two are positive. The middle neuron is the same neuron featured in Figs. 1d–f, 2 and 3. **d** High-power view of Ctip-2-positive neuron indicated with a red rectangular box in 5c. Scale bar 10 µm

Three 400 µm sections containing a total of six FB-positive intracellularly filled neurons with confirmed converging putative synaptic contacts were re-sectioned into 50 µm thick sections and stained for transcription factor Ctip2, a layer V specific marker (Sürmeli et al. 2015). Adjacent sections were stained with cresyl violet and used to delineate the layers of MEC (Fig. 5a). All six neurons were found to be located in sublayer Vb. Five out of six neurons were found to be Ctip2-positive, of which four were classified as multidirectional pyramidal neurons and one classified as a horizontal cell. The latter cell was found to be located on the border between LVa and LVb (leftmost neuron, Fig. 5b–d). The remaining neuron was found to be Ctip2-negative and was classified as a multidirectional pyramidal neuron (rightmost neuron, Fig. 5b, c). This is in line with previous data, indicating that Ctip2-positive neurons in LVb are ones that project to superficial layers of MEC (Ohara et al. 2018).

#### Discussion

Using triple neuroanatomical tracing and confocal imaging 3D reconstruction, we show that axons from RSC and subiculum converge and likely form synaptic contacts on superficially projecting MEC LV principal neurons. Our findings combine the previously reported projections from RSC to LV MEC (Jones and Witter 2007; Czajkowski et al. 2013) with projections from subiculum to LV MEC (van Haeften et al. 1995) by showing that these two major inputs to MEC LV contact the same cells. In addition, we show that the two projections target both dendritic shafts and spines on overlapping portions of the dendritic tree.

### Methodological considerations

Contacts identified with confocal microscopy have previously been compared with synapses characterized with electron microscopy (Cabirol-Pol et al. 2000, Wouterlood Van Haeften et al. 2004). While the confocal method may overestimate the number of contacts compared to EM (Jankowska et al. 1997), it is very likely that at least some of the contacts identified using the confocal method represent true synapses and may provide a reasonable impression of the relative densities and distributions of identified synaptic contacts (Wouterlood et al. 2003; Mishchenko et al. 2010).

Extending a protocol developed previously within our lab (Kononenko and Witter 2012) we stained for synaptic markers to substantiate our claim that putative contacts represent synapses. Synaptophysin is a membrane protein of synaptic vesicles which has been identified as a useful marker for the presence of a presynaptic bouton (Hamos et al. 1989). More than 60% of asymmetric synapses have been shown to express PSD-95 protein at their postsynaptic density (Aoki et al. 2001). PSD-95-staining within dendritic elements of a filled neuron found in close juxtaposition with an axon terminal associated with synaptophysin staining can therefore be used as an indication for a synaptic contact (Wouterlood et al. 2003; Mishchenko et al. 2010). However, since PSD-95 is not necessarily expressed in all synapses (Aoki et al. 2001), these numbers likely represent an underestimation of excitatory inputs. An assessment of PSD-95 and synaptophysin staining has been performed previously for the RSC-to-MEC projection. This technique was then verified with a functional optogenetic in vitro approach as well as with a detailed electron microscopy analysis (Czajkowski et al. 2013).

In the current study, we show similar data for the subicular projection to MEC, although the numbers of contacts that satisfied our criteria were lower for the subicular input than what has been found for the retrosplenial one (Czajkowski et al. 2013). We further know from EM studies that both subicular (van Haeften et al. 1995) and retrosplenial (Sugar Witter et al. 2011) projections terminate on dendritic spines and shafts of neurons in LV of EC. These synapses are predominantly of the asymmetric type, characteristic of excitatory connections (Uchizono 1965). Electrophysiological data also indicate that the direct projection from RSC to MEC LV exerts a powerful excitatory effect (Czajkowski et al. 2013). Likewise, subicular input to the MEC mainly exerts an excitatory influence (Stewart 1997). We, therefore, consider it highly likely that a substantial fraction of contacts of both retrosplenial and subicular inputs identified in our study represent true synapses. It allows drawing conclusions about the functional relationships within the spatial memory circuit. The limits of the applied technique stem from an incomplete labeling of the incoming projections and from possible discrepancies between detected synaptic labelling and true functional synapses. Therefore specific considerations related to the dendritic integration of both inputs and to their relative contribution to MEC LV neuronal firing should be conducted with caution.

### Structure of neurons, distribution of synapses and convergence

The morphological characteristics of LV neurons in our data are in line with previous descriptions (Lingenhohl and Finch 1991; Hamam et al. 2000; Canto and Witter 2012). LV neurons display anatomical and physiological features that suggest an important role in integrating multiple external inputs. Their apical dendrites extend as far as the pial surface and their basal dendrites stretch throughout layer V-VI of MEC, making them potential recipients of inputs that terminate in all layers (Canto et al. 2008). For instance, it has been shown that inputs from parasubiculum (PaS) and presubiculum (PrS) which distribute selectively to layers II or to layer I and III, respectively, contact apical dendrites of LV MEC neurons (Canto et al. 2012).

The latter authors further suggest that such more distal inputs targeting the apical dendrite and its tuft (Canto et al. 2012), have a higher gain and broader temporal window for integration, allowing for effective rate coding (Shadlen and Newsome 1998). We found that retrosplenial and subicular axon fibers contact basal dendrites and the proximal parts of apical dendrites and intermingle relatively close to the soma. The integration of such proximal inputs likely depends on spike timing, and it is likely that they will summate in a linear fashion (Branco and Häusser 2011), though the few instances where we observed the termination of the two in close proximity (< 10 µm) suggest that non-linear summation might occur as well (Takahashi et al. 2012). In summary, a picture emerges where superficially projecting layer V principal neurons differentially integrate several streams of information relevant to spatial processing.

Deep to superficial MEC projections terminate onto dendritic shafts of smooth dendrites of interneurons and onto both dendritic shafts and spines of principal neurons (van Haeften et al. 2003). In all cases, the synapses were predominantly of the asymmetric type. This suggests that the deep to superficial projection in MEC is excitatory, which is corroborated by another study in which it was reported that deep-to-superficial projecting MEC neurons are GABA-negative (Gloveli et al. 2001). This means that both the subiculum and RSC likely exert a disynaptic excitatory effect on the superficial layers of MEC through their projection to the deep layers. This effect is most likely mediated by LVb neurons, given that LVa neurons only contribute a minor projection to superficial layers (Ohara et al. 2018). Our data goes some way to corroborate this, as our retrograde tracer injections in superficial layers mainly labeled cells in deep LV. In addition, five out of six FB-positive neurons were also positive for Ctip2, a marker for LVb neurons (Sürmeli et al. 2015; Ohara et al. 2018). However, we also found some FB-positive cells, apparently located in LVa, which received input from RSC and subiculum. Three out of these neurons were classified as the horizontal morphological type that is believed to mainly reside in Va. One of these neurons, located at the border between LVa and LVb, was found to be Ctip2 positive, so likely belongs to layer Vb as argued recently (Ohara et al. 2021). Our data thus support that LVa neurons that project out of MEC, can contribute to projections to superficial MEC (Czajkowski et al. 2013).

### Relevance to navigation and memory

Adding our current data to previous reports, it appears that LV MEC neurons integrate inputs from HF, PrS and PaS, the areas thought to be essential elements of the main path integration-based navigation system (McNaughton et al. 2006; Moser et al. 2008; Boccara et al. 2010; Canto et al. 2012), and incorporates information from RSC into that stream. Although PrS has been the main structure where head direction neurons are observed, these cells are present in the RSC as well (Chen et al. 1994; Cho and Sharp 2001), and head direction cells of the two regions show similar characteristics in landmark processing (Lozano et al. 2017). They exhibit complex tuning that involves both local and global visual frames (Jacob et al. 2017), as well as vestibular input (Keshavarzi et al. 2021). RSC has strong connections with PrS neurons that in turn influence MEC (Kononenko and Witter 2012). RSC activity has been linked to the recognition and processing of the most permanent landmarks within an environment (Auger et al. 2012), which may point to an important role in attuning grid cell firing to distal cues. This finding is supported by observations in both rats and humans showing that RSC lesions affect a variety of tasks that test different navigational strategies (Vann et al. 2009). The most severe deficits become apparent when animals are forced to change navigational strategies or integrate spatial information of different modularity (Vann et al. 2003; Pothuizen et al. 2008). Furthermore, transient inactivation of the RSC causes the firing of place cells in the hippocampus to become less specific (Cooper and Mizumori 2001). This destabilizing effect on place cell activity is likely to be mediated by the MEC, as the RSC does not project directly to CA3 or CA1 (Sugar Witter et al. 2011).

Importantly, the connectivity of RSC extends beyond the medial temporal lobe and the HPC-MEC circuit. RSC is densely interconnected with the anterior cingulate cortex (Jones et al. 2005) and visual cortex (Fiser et al. 2016; Vélez-Fort et al. 2018, Fischer et al. 2020). RSC also receives inputs from several subcortical structures, including laterodorsal and anterior thalamic nuclei as well as the mammillary bodies (van Groen and Wyss 1992, 1995). These connections convey visceral, proprioceptive and vestibular information (Robertson and Kaitz 1981; Sikes et al. 2008), as well as additional preprocessed spatial input (Clark and Harvey 2016).

This pattern of RSC connectivity and spatial tuning differs in its details from the one described for subiculum (Witter et al. 1990, Kitanishi et al. 2021). It is, therefore, reasonable to speculate that the stream of information fed by RSC into MEC LV is qualitatively different from the hippocampal output provided by subiculum (Balcerek et al. 2021). Recent functional studies indicate that hippocampus and RSC play different but complementary roles in encoding certain spatial features necessary for efficient navigation. Both structures support context recognition, but their relative importance during retrieval is a matter of debate (Cowansage et al. 2014; Tanaka et al. 2014). Their involvement may change upon developing long-term memory. For example, as memory matures, RSC takes over the encoding of goal distance (Patai et al. 2019).

It has been shown that RSC becomes more active as hippocampal independent, schematic memories are formed (Tse et al. 2007, 2011). These observations might be explained within the framework of memory indexing theory (Goode et al. 2020). It has been long postulated (O'Keefe and Dostrovsky 1971; McClelland and Rumelhart 1985), and supported by experimental evidence (Tanaka et al. 2014), that the hippocampus forms an index of dispersed cortical representations of multimodal spatial context. Recent work shows that cortical regions, including RSC or even subcortical structures such as the lateral septum, might also serve an indexing role (Cowansage et al. 2014; Tingley and Buzsáki 2018). To preserve coherent behavior, only one of the indexes must be utilized at a time. When two or more independent memory traces emerge, layer V of MEC would appear as the integrator, and perhaps comparator of the two inputs and allow superficial entorhinal neurons that represent incoming updated information to integrate the optimal selection generated by the index into their submitted information to the hippocampus.

## Supplementary Information

Below is the link to the electronic supplementary material.

Supplementary file1 All reconstructed neurons except cell number 19, which is depicted in Fig. 3. Staining intensity of anterograde tracer injected into subiculum within 300 nm of the reconstructed surface shown color-coded as heat map. Cyan spheres indicate points fulfilling the criteria for a putative synaptic contact with axonal fibers from RSC, while yellow spheres indicate the same for axonal fibers from subiculum. Numbers in right upper corners represent cell numbers corresponding to the X-axis of bar graphs in Fig. 3 c and d. Scale bars 10 µm. Area marked with a dashed red rectangle on image of cell number 22 is depicted in supplementary Fig. 2 (TIF 7084 KB)

Supplementary file2 (TIF 2275 KB)

Supplementary file3 Putative contacts from RSC and Sub intermingle on dendritic segments. Area depicted is marked with a dashed red rectangle in cell number 22 in supplementary Fig. 1. a High power image of Alexa-568-filled dendritic segment (red). Two spines are indicated with cyan and yellow dashed circles. b Alexa-568-filles spine indicated with cyan dashed circle shows overlap with, in this case BDA-positive, bouton from RSC. A different spine, indicated with a yellow dashed circle, overlaps with PHA-L-positive bouton from Sub. The putative contacts are located within 5 µm distance along the dendrite. Scale bar 1 µm (TIF 18267 KB)

## Data Availability

Experimental data will be made available upon reasonable request.
