# Supplementary figures and images for: Retrosplenial and subicular inputs converge on superficially projecting layer V neurons of medial entorhinal cortex
Source: Brain Struct Funct. 2022 Oct 14;227(8):2821–37. doi: 10.1007/s00429-022-02578-8 (PMC9618507; doi:10.1007/s00429-022-02578-8)

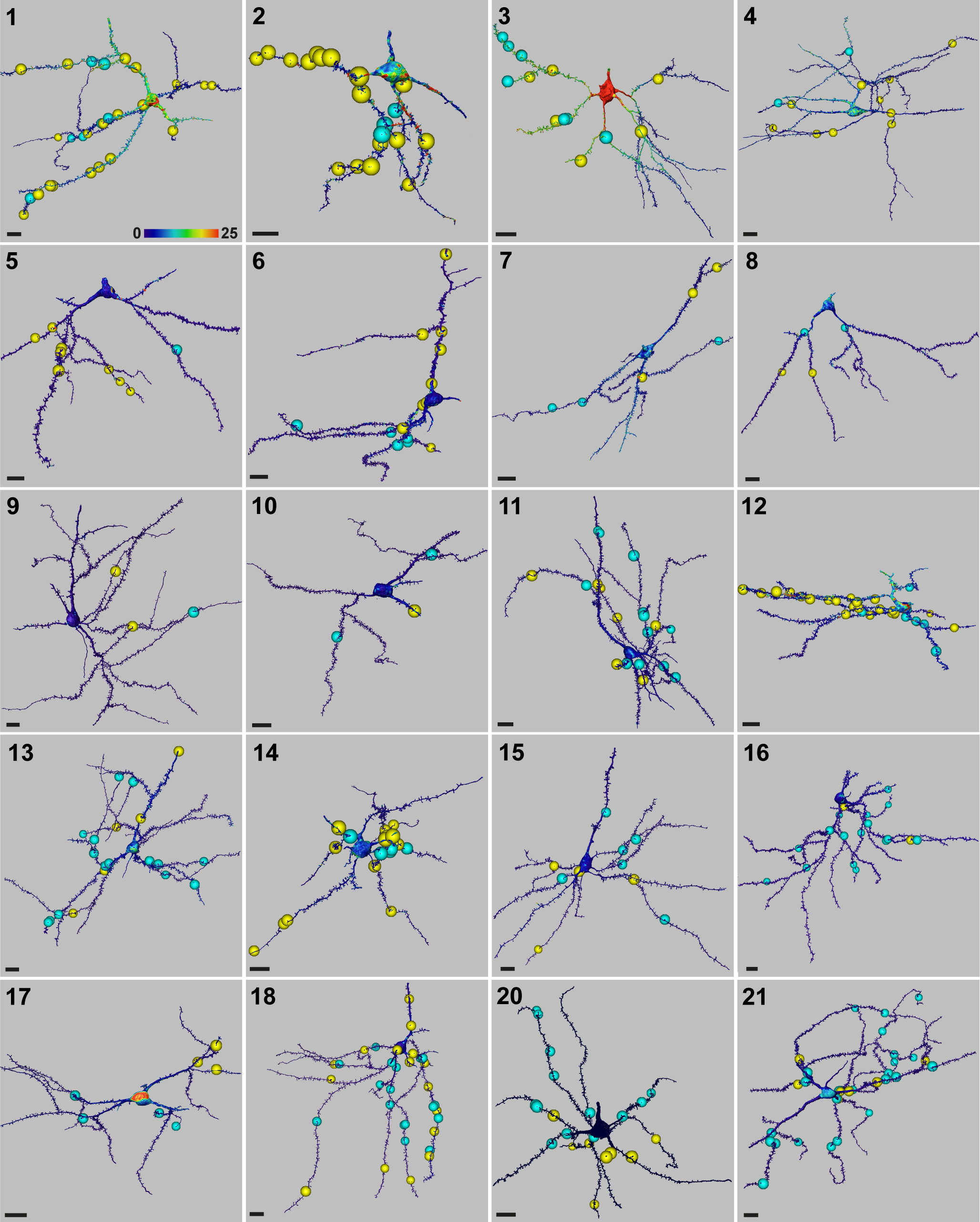

Supplement: Supplementary file 1 — Supplementary file1 All reconstructed neurons except cell number 19, which is depicted in Fig. 3. Staining intensity of anterograde tracer injected into subiculum within 300 nm of the reconstructed surface shown color-coded as heat map. Cyan spheres indicate points fulfilling the criteria for a putative synaptic contact with axonal fibers from RSC, while yellow spheres indicate the same for axonal fibers from subiculum. Numbers in right upper corners represent cell numbers corresponding to the X-axis of bar graphs in Fig. 3 c and d. Scale bars 10 µm. Area marked with a dashed red rectangle on image of cell number 22 is depicted in supplementary Fig. 2 (TIF 7084 KB) [file 429_2022_2578_MOESM1_ESM.tif]

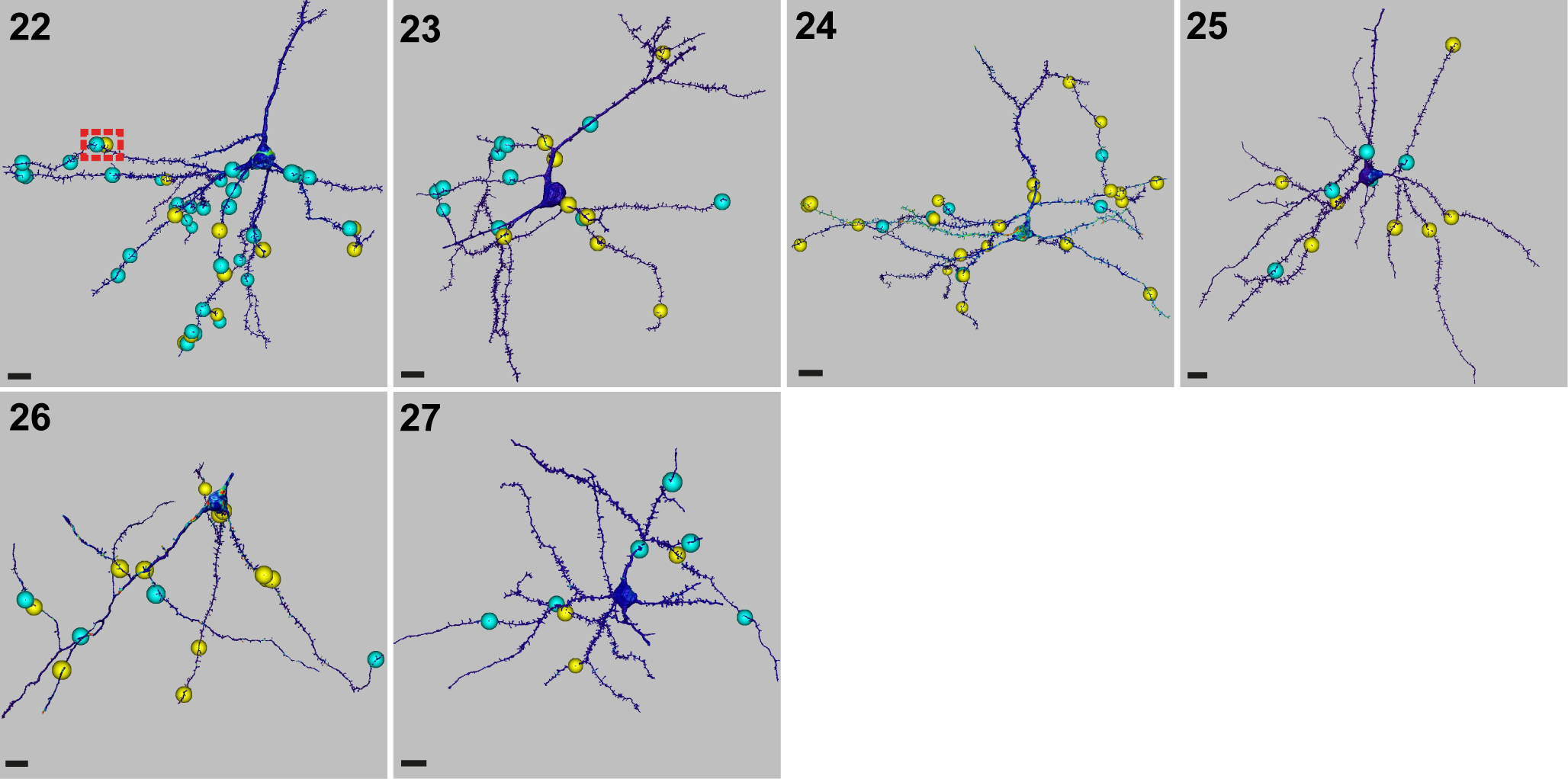

Supplement: Supplementary file 2 — Supplementary file2 (TIF 2275 KB) [file 429_2022_2578_MOESM2_ESM.tif]

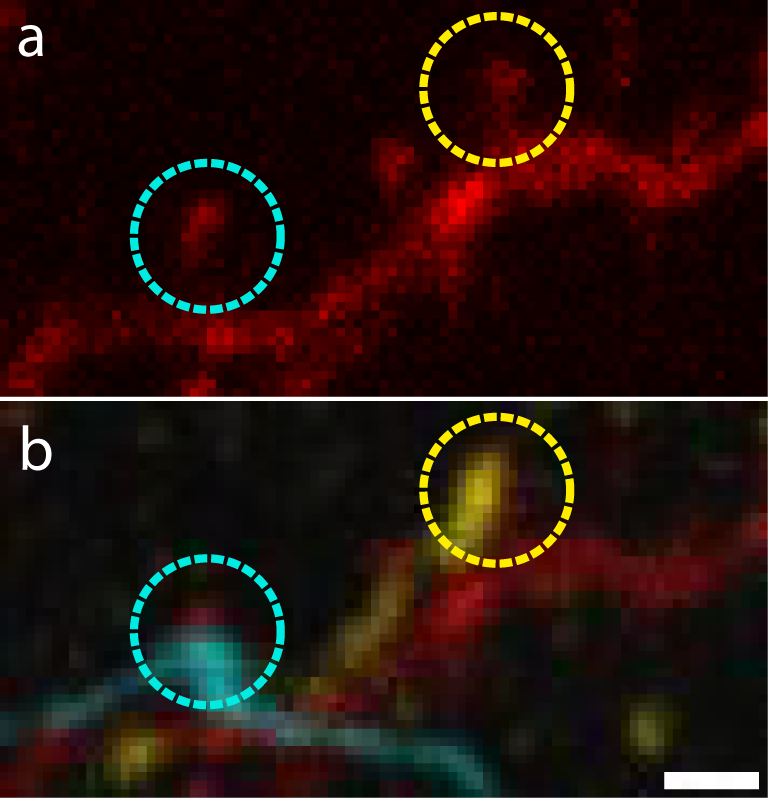

Supplement: Supplementary file 3 — Supplementary file3 Putative contacts from RSC and Sub intermingle on dendritic segments. Area depicted is marked with a dashed red rectangle in cell number 22 in supplementary Fig. 1. a High power image of Alexa-568-filled dendritic segment (red). Two spines are indicated with cyan and yellow dashed circles. b Alexa-568-filles spine indicated with cyan dashed circle shows overlap with, in this case BDA-positive, bouton from RSC. A different spine, indicated with a yellow dashed circle, overlaps with PHA-L-positive bouton from Sub. The putative contacts are located within 5 µm distance along the dendrite. Scale bar 1 µm (TIF 18267 KB) [file 429_2022_2578_MOESM3_ESM.tif]
